# The association between research funding status and clinical research papers’ citation impact in Japan: A cross-sectional bibliometric study

**DOI:** 10.3389/fmed.2022.978174

**Published:** 2022-10-19

**Authors:** Fumito Morisawa, Yuji Nishizaki, Patrick Devos, Naotake Yanagisawa, Kotone Matsuyama, Yasuhiro Homma, Rieko Ueda, Miwa Sekine, Hiroyuki Daida, Tohru Minamino, Shoji Sanada

**Affiliations:** ^1^Department of Cardiovascular Biology and Medicine, Juntendo University Graduate School of Medicine, Tokyo, Japan; ^2^Rare Disease Medical Affairs, Pfizer Japan Inc., Tokyo, Japan; ^3^Division of Medical Education, Juntendo University School of Medicine, Tokyo, Japan; ^4^Medical Technology Innovation Center, Juntendo University, Tokyo, Japan; ^5^Department of Lillometrics, University of Lille, CHU Lille, Lille, France; ^6^Center for Strategic Research Initiative, Nippon Medical School Foundation, Tokyo, Japan; ^7^Department of Health Policy and Management, Nippon Medical School, Tokyo, Japan; ^8^Department of Orthopedic Surgery, Juntendo University School of Medicine, Tokyo, Japan; ^9^Faculty of Health Science, Juntendo University, Tokyo, Japan; ^10^Clinical and Translational Research Center, Kobe University Hospital, Kobe, Japan

**Keywords:** clinical research, research funding, citation impact, SIGAPS, category normalized citation impact, bibliometrics

## Abstract

**Introduction:**

Studies have not sufficiently clarified the differences in citation impact between funded and non-funded clinical research papers. Hence, this study seeks to evaluate the relation between research funding status and clinical research papers’ citation impact in different research fields using multiple evaluation indices.

**Methods:**

In this cross-sectional bibliometric study, clinical research papers published by core clinical research hospitals in Japan were compared retrospectively in terms of times cited (TC), category normalized citation impact (CNCI), citation percentile (CP), journal impact factor (JIF), the Software to Identify, Manage, and Analyze Scientific Publications (SIGAPS) category, and whether they were the funded clinical research. The association between research funding status or the SIGAPS category and CNCI ≥ 2 was analyzed using logistic regression analysis.

**Results:**

11 core clinical research hospitals published 553 clinical research papers, of which 120 were non-funded and 433 were funded (public institution-funded and industry-funded). The study found that funded clinical research papers (public institution-funded and industry-funded) had significantly higher TC, CNCI, CP, and JIF than non-funded ones [TC: 8 (3–17) vs. 14 (8–31), *p* < 0.001; CNCI: 0.53 (0.19–0.97) vs. 0.87 (0.45–1.85), *p* < 0.001; CP: 51.9 (24.48–70.42) vs. 66.7 (40.53–88.01), *p* < 0.001; JIF: 2.59 (1.90–3.84) vs. 2.93 (2.09–4.20) *p* = 0.008], while the proportion of A or B rank clinical research papers of the SIGAPS category was not significantly different between the two groups (30.0 vs. 34.9%, *p* = 0.318). In the logistic regression analysis, having a CNCI ≥ 2 was significantly associated with research funding (public institution-funded and industry-funded) and publication in A or B rank journals of the SIGAPS category [research funding: Estimate 2.169, 95% confidence interval (CI) 1.153–4.083, *p* = 0.016; SIGAPS category A/B: Estimate 6.126, 95% CI 3.889–9.651, *p* < 0.001].

**Conclusion:**

Analysis *via* multiple indicators including CNCI and the SIGAPS category, which allows for a comparison of the papers’ citation impact in different research fields, found a positive relation between research funding status and the citation impact of clinical research papers.

## Introduction

It is important to explain the differences in citation impact between funded and non-funded clinical studies. Scholars have reported that industry-funded clinical studies tend to be published in journals with a higher journal impact factor (JIF) (1–4), which has been used to evaluate a research paper’s citation impact. However, the drawback to JIF, which is necessary to understand, is that its values cannot be compared across research fields because the frequency and tendency of citations differ depending on the field (5–7). In fact, for example, several journals of cardiac and cardiovascular systems in the Web of Science category tend to have a higher overall JIF than top journals in a particular category (8), and it is difficult to compare the JIF between journals in different clinical research disciplines.

The number of citations [or times cited (TC)] is also a well-known evaluation index for a research paper’s citation impact, but it is influenced by the research field, publication year, and paper category (5). Such a weakness is, therefore, compensated by citation percentile (CP) and category normalized citation impact (CNCI). Studies have reported that papers with different publication years and research fields can be compared (9–11), which highlights the importance of evaluating the relation between research funding status and clinical research papers’ citation impact using multiple evaluation indices.

The new evaluation index has been attracting attention and being used in recent years. The Software to Identify, Manage, and Analyze Scientific Publications (SIGAPS) scoring system is known as an evaluation index of citation impact for papers that compensate for the shortcomings of JIF (12, 13). The SIGAPS scoring system, which was developed in France, has been recognized by the French government as an extremely reliable tool (14). Hence, each research institute in France is officially funded based on an indicator calculated using the SIGAPS scoring system (15). The SIGAPS scoring system classifies each journal into specific fields (Web of Science categories) based on the Thomson Institute for Scientific Information, ranks them from high to low JIF, and assigns them to their own categories. Simply put, the SIGAPS category can be considered the relative JIF, which makes it possible to compare the impact factor of journals among different research fields in medical research (14–16). In SIGAPS, journals ranked A or B are overweighted because they correspond to the 25% of journals with the highest JIF in each category (Q1 journals in Journal Citation Reports) (14, 15, 17). In France, for a given institution, the percentage of articles published in A or B journals is used as an indicator of performance.

One study reported that funded phase 3 trials were associated with neither publication in journals with higher JIF nor citation (18), and other studies found that funded research was not necessarily associated with citation (19, 20). No reports have investigated the relation between research funding status and clinical research papers’ citation impact in different research fields of medical research from various aspects using multiple evaluation indices, including CNCI and the SIGAPS category. Therefore, we focused on the evaluation by multiple indices to clarify whether the research funding status influences the clinical research papers’ citation impact. Although JIF has some drawbacks, it is still one of the widely used bibliometric index, and in addition to the bibliometric indices, such as the CNCI and SIGAPS categories, we added JIF for this study. This study aims to (1) compare the bibliometric indexes of articles (TC, CNCI, CP, JIF, and the SIGAPS category) from funded and unfunded studies and (2) determine if it is possible to identify factors that could explain why an article is frequently cited.

## Materials and methods

This study was a retrospective cross-sectional bibliometric study, and we focused on all clinical research papers in English published by all core clinical research hospitals in Japan approved by the Ministry of Health, Labor and Welfare (MHLW) in November 2018, and TC, CP, CNCI, JIF, and the SIGAPS category were aggregated retrospectively according to whether they constituted funded clinical research. In addition, the relation between research funding status or the SIGAPS category and CNCI was examined.

All core clinical research hospitals have submitted their all-clinical research papers to the MHLW since the last 3 years, which is one of the requirements for approval as a core clinical research hospital; all these papers were put in the fiscal year business report of the MHLW website. At the time of the data collection, the latest fiscal year business report publicly available on the MHLW website was for fiscal year 2017 (21). We collected data on all the clinical research papers of each hospital from the fiscal year 2017 business report on the MHLW website. All the papers had been published in journals from June 2013 to March 2017 (21). The following categories were noted: type of clinical trial (e.g., drug development or medical device), study design (e.g., randomized controlled trial or pilot study), and publishing options (e.g., open access, Web of Science). This information is summarized in Supplementary material.

Data on the research funding status for these papers were gathered based on their Acknowledgments and any funding information section. Research funding status was classified as (1) non-funded, (2) public institution-funded, and (3) industry-funded. The first category, non-funded, was defined as papers that did not mention any research funding. Meanwhile, the second and third categories comprised papers that had received funding. The public institution-funded category contained papers that mentioned having received research funding from a public institution, such as administrative agency, excluding private companies. The industry-funded category contained papers that mentioned having received research funding from a private company, such as a pharmaceutical agent. The bibliometric indices of each paper (TC, CNCI, CP, JIF, and the SIGAPS category) were obtained from the InCites Benchmarking & Analytics™ (Clarivate™) (22) and SIGAPS as of July 2021.

### Core clinical research hospitals

Core clinical research hospitals in Japan are stipulated in the Medical Care Act, and hospitals that meet certain requirements, such as publishing 45 or more high-quality clinical trial papers in the last 3 years, are approved by the Minister of Health, Labor and Welfare. These hospitals play a pivotal role in international-standard clinical research and investigator-initiated clinical trials with the aim to develop innovative medicines and medical devices originating from Japan. When we planned this study, 11 medical institutions in Japan have been approved (eight national university hospitals, two national centers, and one private university hospital) since this system became effective in April 2015 (15, 21). Nine medical institutions are located in the 23 wards in Tokyo or 20 cities designated by the Ministry of Internal Affairs and Communications and two medical institutions are located in provincial cities (21). The median number of beds in each hospital was 1,044 (minimum–maximum: 425–1,275) (21). According to a Times Higher Education report, two of these core clinical research hospitals are among the global top 100 high-research-performance universities (23).

### Times cited

The number of citations was obtained from the Web of Science Core Collection, considering the age of the article and its disciplinary field.

### Citation percentile calculation method

A publication’s CP is calculated by sorting all the publications in a particular year and within the same disciplinary field (Web of Science category) in descending order of the number of citations and then computing their percentiles. If the CP is above 90, the paper is one of the top 10% most cited in this Web of Science category; accordingly, if the CP is above 99, the paper is a part of the top 1% most cited in this Web of Science category (9).

### Category normalized citation impact calculation method

For a given publication, CNCI is calculated by dividing the observed number of citations by the expected number of citations (mean citation count of all papers of a document type published the same year in the same research field). If the paper belongs to more than one research field, the average of the actual-to-expected-citation ratio for each research field is used. A CNCI value of 1 indicates equivalence to the world average; if the CNCI value is more than 1, the paper is considered to be cited above the world average; and if the CNCI value is less than 1, the paper is considered to be cited below the world average. If the CNCI value is 2, the paper is considered to be cited twice the world average (9). The threshold of 2 is often used to identify high impact publications in terms of citations.

### The software to identify, manage, and analyze scientific publications category calculation method

SIGAPS categories are based on the impact factor of journals provided by the Journal Citation Reports (Clarivate). For each disciplinary field (Web of Science categories), journals are ranked according to decreasing impact factor. The quartiles and the 90th percentile are then used to calculate the SIGAPS category (A to E) or NC (i.e., non-classified because it is not indexed in the Journal Citation Reports). The journal rankings according to the JIF percentile are as follows: the 90th and above percentile is the A rank, the 75th–90th percentile is the B rank, the 50th–75th percentile is the C rank, the 25th–50th percentile is the D rank, and the 25th and below percentile is the E rank (13–16). In the SIGAPS score, A rank journals are weighted 8 points, B rank journals 6 points, C rank journals 4 points, D rank journals 3 points, and E rank journals 2 points. Journals without JIF (NC category) are weighted 1 point (14, 15).

### Statistical analysis

First, descriptive analyses of each index (TC, CNCI, CP, JIF, and the SIGAPS category) were performed to examine and review the data. Assumption of normality was assessed using the Shaphiro-Wilk test. Bibliometric indexes were compared according to funding status. According to asymmetric distributions, numerical parameters were compared using non-parametric tests (Wilcoxon tests in the case of two groups, and Kruskall–Wallis test in the case of three groups). Multiple comparisons were performed using a Type I error correction using the Dwass, Steel and Critchlow-Fligner method. Two article subgroups were also defined: CNCI ≥ 2 and CNCI < 2. Moreover, they were compared according to funding status and the SIGAPS category (A or B vs. C, D, or E) using the Chi-square test or Fisher’s exact test as appropriate. Finally, a logistic regression was run with CNCI ≥ 2 as the dependent variable and research funding status (funded vs. non-funded) and the SIGAPS category (A or B vs. C, D, or E) as independent variables. A *p*-value < 0.05 was considered statistically significant. Statistical analysis was performed using the Statistical Analysis System Software V9.4 (Cary, NC, USA).

## Results

In the fiscal year 2017 business report on the MHLW website, it was reported that the Minister of Health, Labor and Welfare approved 11 core clinical research hospitals. These hospitals published 571 clinical research papers in English from June 2013 to March 2017, and the fields of study for the clinical research papers were oncology, gastroenterology and hepatology, pharmacology and pharmacy, urology and nephrology, medicine, research and experimental, cardiac and cardiovascular systems, clinical neurology, respiratory system, ophthalmology, peripheral vascular disease, and others. Among 571 clinical research papers, there were 419 (73.4%) drug clinical research, 244 (42.7%) randomized controlled trials, 64 (11.2%) pilot studies, and 422 (73.9%) published in open access journals. The characteristics of each clinical research shown by the status of research funding can be found in Supplementary Table 1.

The 553 clinical research papers, excluding 18 papers that cannot be calculated or classified *via* the SIGAPS scoring system, were analyzed. The median number of clinical research papers published by each hospital was 48 (minimum–maximum: 41–65). There were a total of 120 non-funded clinical research papers, 225 public institution-funded clinical research papers, and 208 industry-funded clinical research papers. The median or interquartile range of each index in all clinical research papers is shown in Table 1. In the analysis of TC, CNCI, CP, and JIF in terms of whether or not they were funded clinical papers, a statistically significant difference was observed between funded clinical research papers (public institution-funded and industry-funded) and non-funded ones [TC: 8 (3–17) vs. 14 (8–31), *p* < 0.001; CNCI: 0.53 (0.19–0.97) vs. 0.87 (0.45–1.85), *p* < 0.001; CP: 51.9 (24.48–70.42) vs. 66.7 (40.53–88.01), *p* < 0.001; JIF: 2.59 (1.90–3.84) vs. 2.93 (2.09–4.20) *p* = 0.008]. Concerning the SIGAPS category, no statistically significant differences was found between the two groups in the proportion of A or B rank clinical research papers (30.0 vs. 34.9%, *p* = 0.318) (Table 1).

**TABLE 1:** Each index according to research funding status.

	All research	Research funding status		Non-funded vs. funded
		
		Non-funded research	Funded research^a^	
	*n* = 553	*n* = 120	*n* = 433	*p*-value
TC	13 (6–28)	8 (3–17)	14 (8–31)	<0.001
CNCI	0.80 (0.37–1.69)	0.53 (0.19–0.97)	0.87 (0.45–1.85)	<0.001
CP	63.94 (36.42–85.75)	51.9 (24.48–70.42)	66.7 (40.53–88.01)	<0.001
JIF	2.82 (2.02–4.12)	2.59(1.90–3.84)	2.93 (2.09–4.20)	0.008
SIGAPS category				
A/B	187 (33.8)	36 (30.0)	151 (34.9)	0.318
C/D/E	366 (66.2)	84 (70.0)	282 (65.)	

Values are presented as number (percentage) or median (interquartile range). ^a^Funded research includes public institution- and industry-funded. TC, Times cited; CNCI, Category Normalized Citation Impact; CP, Citation percentile; JIF, Journal impact factor; SIGAPS, Classification based on Impact Factors adjusted by disciplinary field.

Table 2 summarizes the analysis of each bibliometric index according to the three research funding status groups. A statistically significant difference among the three groups was observed in the numerical indicators [TC: 8 (3–17) vs. 13 (7–30) vs. 15 (8–36), *p* < 0.001; CNCI: 0.53 (0.19–0.97) vs. 0.85 (0.42–1.80) vs. 0.89 (0.49–2.02), *p* < 0.001; CP: 51.94 (24.48–70.42) vs. 64.29 (39.88–87.02) vs. 68.23 (46.31–88.75), *p* < 0.001; JIF: 2.59 (1.90–3.84) vs. 3.08 (1.91–4.41) vs. 2.92 (2.29–3.97), *p* = 0.028]. *Post hoc* tests demonstrated that a statistically significant difference was observed between public institution-funded clinical research papers and non-funded ones [TC: 8 (3–17) vs. 13 (7–30), *p* < 0.001; CNCI: 0.53 (0.19–0.97) vs. 0.85 (0.42–1.80), *p* < 0.001; CP: 51.94 (24.48–70.42) vs. 64.29 (39.88–87.02), *p* < 0.001; JIF: 2.59 (1.90–3.84) vs. 3.08 (1.91–4.41), *p* = 0.078] and between industry-funded clinical research papers and non-funded ones [TC: 8 (3–17) vs. 15 (8–36), *p* < 0.001; CNCI: 0.53 (0.19–0.97) vs. 0.89 (0.49–2.02), *p* < 0.001; CP: 51.94 (24.48–70.42) vs. 68.23 (46.31–88.75), *p* < 0.001; JIF: 2.59 (1.90–3.84) vs. 2.92 (2.29–3.97), *p* = 0.021], but that no statistically significant differences existed between public institution-funded clinical research papers and industry-funded ones [TC: 13 (7–30) vs. 15 (8–36), *p* = 0.512; CNCI: 0.85 (0.42–1.80) vs. 0.89 (0.49–2.02), *p* = 0.737; CP: 64.29 (39.88–87.02) vs. 68.23 (46.31–88.75), *p* = 0.657; JIF: 3.08 (1.91–4.41) vs. 2.92 (2.29–3.97), *p* = 0.958]. Finally, no statistically significant difference among the three groups was observed in the proportion of A or B rank clinical research papers of the SIGAPS category [30.0 vs. 38.2 vs. 31.3%, *p* = 0.188].

**TABLE 2:** Comparison of each index according to research funding status group.

	Research funding status				Non-funded vs. public institution	Non-funded vs. industry	Public institution vs. industry
				
	Non-funded (*n* = 120)	Public institution-funded (*n* = 225)	Industry-funded (*n* = 208)	Overall *p*-value	*p*-value	*p*-value	*p*-value
TC	8 (3–17)	13 (7–30)	15 (8–36)	<0.001	<0.001	<0.001	0.512
CNCI	0.53 (0.19–0.97)	0.85 (0.42–1.80)	0.89 (0.49–2.02)	<0.001	<0.001	<0.001	0.737
CP	51.94 (24.48–70.42)	64.29 (39.88–87.02)	68.23 (46.31–88.75)	<0.001	<0.001	<0.001	0.657
JIF	2.59 (1.90–3.84)	3.08 (1.91–4.41)	2.92 (2.29–3.97)	0.028	0.078	0.021	0.958
**SIGAPS category**							
A/B	36 (30.0)	86 (38.2)	65 (31.3)	0.188	No pairwise comparisons		
C/D/E	84 (70.0)	139 (61.8)	143 (68.7)				

Values are presented as a number (percentage) or median (interquartile range). TC, Times cited; CNCI, Category Normalized Citation Impact; CP, Citation percentile; JIF, Journal impact factor; SIGAPS, Classification based on Impact Factors adjusted by disciplinary field.

Finally, concerning the predictive factors of highly cited clinical research papers (CNCI ≥ 2), two significant factors were identified in univariate analysis (Chi-square test): research funding (public institution-funded and industry-funded) (X^2^ = 6.749, *p* = 0.009) and publication in A or B rank journals of the SIGAPS category (X^2^ = 70.689, *p* < 0.001). The proportion of clinical research papers with a CNCI ≥ 2 did not differ between public institution-funded clinical research papers and industry-funded ones (20.0 vs. 25.0%, *p* = 0.213). Table 3 shows that, in univariate analysis, a clinical research paper that received research funding (public institution-funded and industry-funded) was approximately twice as likely to be among the clinical research papers with a CNCI ≥ 2. A clinical research paper published in A or B rank journals of the SIGAPS category (journals ranked Q1 in Journal Citation Reports by Clarivate) was approximately six times more likely to be among the clinical research papers with CNCI ≥ 2.

**TABLE 3:** Parameters associated to CNCI ≥ 2 (univariate analysis).

Parameter	Estimate	95% confidence limits		*p*-value
Research funding^a^	2.186	1.198	3.988	0.006
SIGAPS category A/B	6.138	3.908	9.641	<0.001

^a^Research funding includes public institution- and industry-funded. CNCI, Category Normalized Citation Impact; SIGAPS, Classification based on Impact Factors adjusted by disciplinary field.

In multivariate logistic regression, the parameters of both research funding (public institution-funded and industry-funded) and publication in A or B rank journals of the SIGAPS category remained significantly associated with CNCI ≥ 2 [research funding: Estimate 2.169, 95% confidence interval (CI) 1.153–4.083, *p* = 0.016; SIGAPS category A/B: Estimate 6.126, 95% CI 3.889–9.651, *p* < 0.001]. This result was to be expected as the previous analysis had shown that there was no significant relationship between research funding (public institution-funded and industry-funded) and publication in A or B rank journals of the SIGAPS category (Table 4).

**TABLE 4:** Parameters associated to CNCI ≥ 2 (multivariate analysis).

Parameter	Estimate	95% confidence limits		*p*-value
Research funding^a^	2.169	1.153	4.083	0.016
SIGAPS category A/B	6.126	3.889	9.651	<0.001

^a^Research funding includes public institution- and industry-funded. CNCI, Category Normalized Citation Impact; SIGAPS, Classification based on Impact Factors adjusted by disciplinary field.

## Discussion

The present research found that funded clinical research papers (public institution-funded and industry-funded) were high citation impact studies. This study used an unprecedented CNCI and the SIGAPS category alongside TC, CP, and JIF to evaluate clinical research papers’ citation impact. This is because JIF cannot be compared across fields, as the frequency and tendency of citations vary depending on the research area (5–7), a drawback shared by the TC (5). Therefore, we deemed it necessary to assess clinical research papers’ citation impact from various aspects using multiple evaluation indices such as CNCI and the SIGAPS category, which allows for the comparison of the papers’ citation impact in different research fields (9, 11, 14–16). While scholars have reported an association between research funding and JIF and between research funding and TC (1, 24, 25), to the best of our knowledge, no studies have examined the relation between research funding and multiple indices. Therefore, this study is the first to report a link between research funding and clinical research papers using multiple indices such as CNCI and the SIGAPS category.

We also showed a significant association between research funding (public institution-funded and industry-funded) and CNCI ≥ 2. Because a paper with a CNCI of 2 is considered to be cited twice the world average, we analyzed the funding types for highly cited papers with CNCI ≥ 2. Industry-funded research is often introduced at academic conferences and symposiums, and the increase in citations may be caused by these public-relations efforts to disseminate clinical research findings (1). In addition, these projects have generally been widely evaluated, and only the best ones receive funding. This may also explain why they are cited more frequently. Additionally, since this study focused on clinical research papers reported by core clinical research hospitals, it also included physician-led clinical trials of pharmaceutical agents. In fact, three-quarters of industry-funded research papers in this study were clinical trials funded by pharmaceutical agents, and it is possible that sufficient funding has been directed to high-quality clinical trials. Moreover, these clinical trials are conducted in compliance with Good Clinical Practice, so the research may be high-quality and its results highly reliable when compared to research that is not conducted in compliance with Good Clinical Practice. Therefore, these papers may attract attention and may be cited in many papers consequently.

The CNCI and the impact factor (thus the SIGAPS category) are directly related to the number of citations of clinical research papers. It is therefore normal to find a relationship between CNCI ≥ 2 and the fact that a clinical research paper is published in A or B rank journals of the SIGAPS category. More surprisingly, we showed that even after adjusting for this parameter, the difference between funded (public institution-funded and industry-funded) and non-funded clinical research papers remained significant. This means that funded clinical research papers, even if they are not published in high impact journals, can be highly cited (26).

We believe that research funding improves the citation impact of clinical research papers as follows. Conducting clinical research requires a wide range of tasks such as support for the preparation of documents for submission to the institutional review board, patient enrollment, data management, and statistical analysis, which are costly and time-consuming (27). Because investigators are usually busy, they often find it difficult to perform these tasks by themselves, which necessitates the establishment of a research implementation system consisting of clinical research support including a research secretariat, a clinical research coordinator (CRC), and a data manager. In phase 3 clinical trials, labor accounted for 37% of total costs, and outsourcing accounted for 22% (28). Clinical trials encounter challenges such as cost and staff shortages (29, 30), and these barriers are the same for investigator- and industry-initiated clinical trials (29). In addition, the electronic data capture (EDC) system may reduce data collection time using a paper case report form (CRF) (31), and it is further suggested that the EDC will improve data collection accuracy (32). However, implementing the EDC system requires a high initial investment cost and technical support (33). Substantial expense is also needed to establish the research implementation system, which includes employing a staff and operating the data management system, and we believe that research funding is significantly linked to the improvement of the citation impact of clinical research.

Patient enrollment is also a vital aspect in the success of clinical research; however, only 31% of clinical trials have achieved their target patient count. In addition, approximately 45% of clinical trials have enrolled less than 80% of their target number of patients (34), making patient enrollment a barrier to clinical trial success (29, 30). Collaboration with clinical research assistants such as CRCs is indispensable for efficient patient enrollment and requires funding. Simply put, research funding and the improvement of clinical research papers’ citation impact are closely related.

This study has several limitations. First, open-access journals may have influenced the findings. Because of the spread of open-access journals in recent years, subscription journals and open-access journals may be different in terms of number of views and citations (35). Here, the influence of subscription journals and open-access journals has not been addressed, and the increase in the number of open-access journals may have influenced each index. Second, this study did not address self-citation issues, and self-citation may have influenced the citation impact of clinical research papers. Third, this study did not consider the study design of each clinical research, i.e., whether they are single-centered or multicentered, RCTs or non-RCTs and so on, which may influence the adopted journals and the citation count. Fourth, this study did not consider the causal relationship. In particular, we did not consider that the most impactful work is selected for funding and funding makes impactful work possible because of retrospective cross-sectional study design. Fifth, in this study, non-funded was defined as not having received research funding; i.e., there was no mention of funding in the Acknowledgments or a funding information section. Therefore, even if the research funding status of the papers was not mentioned directly, the research may have received funding from an entity other than a public institution or industry. Sixth, this study did not investigate useful evaluation indices, such as collaboration impact, relative citation ratio (RCR), and h-index. In the future, we will try to conduct another research project that uses these evaluation indices. The final issue concerns generalizability; as this study included only 553 clinical research papers published by 11 core clinical research hospitals in Japan, the results cannot be applied to clinical research in general.

## Conclusion

This study found a positive association between research funding status and clinical research papers’ citation impact after an evaluation that used multiple indicators including CNCI and the SIGAPS category, which allows for a comparison of the papers’ citation impact in different research fields.

## Data availability statement

The data that support the findings of this study are available from Clarivate (https://clarivate.com/); however, restrictions apply regarding the availability of these data, which were used under license for the current study and are not publicly available.

## Author contributions

FM and YN contributed to conceptualization. FM, YN, PD, and RU contributed to data curation and investigation. FM, YN, PD, and NY contributed to formal analysis. SS contributed to funding acquisition. YN contributed to project administration. PD contributed to resources. HD, TM, and SS contributed to supervision. FM, YN, and PD contributed to original draft preparation. All authors contributed to review, editing, and reviewed and approved the final manuscript for submission.
