# Supplementary material for: The association between research funding status and clinical research papers’ citation impact in Japan: A cross-sectional bibliometric study
Source: Front Med (Lausanne). 2022 Oct 19;9:978174. doi: 10.3389/fmed.2022.978174 (PMC9626813; doi:10.3389/fmed.2022.978174)
Supplement: Supplementary file 1 [file Table_1.DOCX]

Supplementary Material

# Supplementary Table 1: Characteristics of clinical trials

|  |  | Research funding status | | | |
| --- | --- | --- | --- | --- | --- |
|  |  | All research (n = 571) | Non-funded (n = 125) | Public institution-funded (n = 236) | Industry-funded (n = 210) |
| Type of clinical trial |  |  |  |  |  |
| Drug |  | 419(73.4) | 70(56.0) | 158(66.9) | 191(91.0) |
|  | Phase Ⅰ | 73(17.4) | 6(8.6) | 14(8.9) | 53(27.7) |
|  | Phase Ⅰ/Ⅱ | 16(3.8) | 1(1.4) | 6(3.8) | 9(4.7) |
|  | Phase Ⅱ | 116(27.7) | 27(38.6) | 52(32.9) | 37(19.4) |
|  | Phase Ⅱ/Ⅲ | 15(3.6) | 0(0.0) | 6(3.8) | 9(4.7) |
|  | Phase Ⅲ | 69(16.5) | 3(4.3) | 22(13.9) | 44(23.0) |
|  | Phase Ⅳ | 12(2.9) | 1(1.4) | 4(2.5) | 7(3.7) |
|  | Others^a^ | 118(28.2) | 32(45.7) | 54(34.2) | 32(16.8) |
| Diagnosis |  | 23(4.0) | 5(4.0) | 14(5.9) | 4(1.9) |
| Medical device |  | 70(12.3) | 26(20.8) | 34(14.4) | 10(4.8) |
| Surgical procedures |  | 24(4.2) | 14(11.2) | 9(3.8) | 1(0.5) |
| Supplement |  | 4(0.7) | 3(2.4) | 1(0.4) | 0(0.0) |
| Regenerative medical products |  | 14(2.5) | 3(2.4) | 8(3.4) | 3(1.4) |
| Others^b^ |  | 17(3.0) | 5(3.2) | 12(5.1) | 1(0.5) |
| Study design |  |  |  |  |  |
| Randomized controlled trial |  | 244(42.7) | 46(36.8) | 102(43.2) | 96(45.7) |
| Non-Randomized controlled trial |  | 327(57.3) | 79(63.2) | 134(56.8) | 114(54.3) |
| Pilot study |  | 64(11.2) | 17(13.6) | 23(9.7) | 24(11.4) |
| Non-pilot study |  | 507(88.8) | 108(86.4) | 213(90.3) | 186(88.6) |
| Single-center |  | 227(39.8) | 67(53.6) | 107(45.3) | 53(25.2) |
| Multicenter |  | 344(60.2) | 58(46.4) | 129(54.7) | 157(74.8) |
| Publishing options |  |  |  |  |  |
| Open access |  | 422(73.9) | 80(64.0) | 172(72.9) | 170(81.0) |
| Non open access |  | 149(26.1) | 45(36.0) | 64(27.1) | 40(19.0) |
| Web of Science categories |  |  |  |  |  |
| Allergy |  | 2(0.4) | 0(0.0) | 0(0.0) | 2(1.0) |
| Anesthesiology |  | 2(0.4) | 2(1.6) | 0(0.0) | 0(0.0) |
| Biochemistry & Molecular Biology |  | 2(0.4) | 0(0.0) | 2(0.8) | 0(0.0) |
| Biotechnology & Applied Microbiology |  | 1(0.2) | 0(0.0) | 1(0.4) | 0(0.0) |
| Cardiac & Cardiovascular Systems |  | 31(5.4) | 10(8.0) | 12(5.1) | 9(4.3) |
| Cell & Tissue Engineering |  | 1(0.2) | 0(0.0) | 1(0.4) | 0(0.0) |
| Cell Biology |  | 3(0.5) | 0(0.0) | 2(0.8) | 1(0.5) |
| Chemistry |  | 1(0.2) | 0(0.0) | 1(0.4) | 0(0.0) |
| Clinical Neurology |  | 16(2.8) | 2(1.6) | 8(3.4) | 6(2.9) |
| Critical Care Medicine |  | 1(0.2) | 0(0.0) | 0(0.0) | 1(0.5) |
| Dentistry, Oral Surgery & Medicine |  | 5(0.9) | 1(0.8) | 3(1.3) | 1(0.5) |
| Dermatology |  | 8(1.4) | 0(0.0) | 2(0.8) | 6(2.9) |
| Endocrinology & Metabolism |  | 14(2.5) | 3(2.4) | 6(2.5) | 5(2.4) |
| Gastroenterology & Hepatology |  | 45(7.9) | 14(11.2) | 12(5.1) | 19(9.0) |
| Geriatrics & Gerontology |  | 4(0.7) | 0(0.0) | 2(0.8) | 2(1.0) |
| Gerontology |  | 2(0.4) | 1(0.8) | 1(0.4) | 0(0.0) |
| Health Care Sciences & Services |  | 1(0.2) | 0(0.0) | 1(0.4) | 0(0.0) |
| Hematology |  | 9(1.6) | 1(0.8) | 2(0.8) | 6(2.9) |
| Immunology |  | 7(1.2) | 1(0.8) | 5(2.1) | 1(0.5) |
| Infectious Diseases |  | 3(0.5) | 1(0.8) | 0(0.0) | 2(1.0) |
| Integrative & Complementary Medicine |  | 4(0.7) | 1(0.8) | 2(0.8) | 1(0.5) |
| Medicine, General & Internal |  | 8(1.4) | 0(0.0) | 6(2.5) | 2(1.0) |
| Medicine, Research & Experimental |  | 33(5.8) | 13(10.4) | 14(5.9) | 6(2.9) |
| Microbiology |  | 1(0.2) | 1(0.8) | 0(0.0) | 0(0.0) |
| Multidisciplinary Sciences |  | 7(1.2) | 1(0.8) | 6(2.5) | 0(0.0) |
| Neurosciences |  | 5(0.9) | 0(0.0) | 5(2.1) | 0(0.0) |
| Nutrition & Dietetics |  | 2(0.4) | 0(0.0) | 1(0.4) | 1(0.5) |
| Obstetrics & Gynecology |  | 3(0.5) | 2(1.6) | 0(0.0) | 1(0.5) |
| Oncology |  | 155(27.1) | 23(18.4) | 73(30.9) | 59(28.1) |
| Ophthalmology |  | 20(3.5) | 6(4.8) | 9(3.8) | 5(2.4) |
| Orthopedics |  | 3(0.5) | 0(0.0) | 2(0.8) | 1(0.5) |
| Otorhinolaryngology |  | 5(0.9) | 0(0.0) | 4(1.7) | 1(0.5) |
| Peripheral Vascular Disease |  | 14(2.5) | 2(1.6) | 4(1.7) | 8(3.8) |
| Pharmacology & Pharmacy |  | 47(8.2) | 7(5.6) | 9(3.8) | 31(14.8) |
| Physiology |  | 1(0.2) | 0(0.0) | 1(0.4) | 0(0.0) |
| Psychiatry |  | 10(1.8) | 1(0.8) | 6(2.5) | 3(1.4) |
| Psychology, Clinical |  | 2(0.4) | 1(0.8) | 1(0.4) | 0(0.0) |
| Radiology, Nuclear Medicine & Medical Imaging |  | 14(2.5) | 3(2.4) | 9(3.8) | 2(1.0) |
| Rehabilitation |  | 3(0.5) | 1(0.8) | 1(0.4) | 1(0.5) |
| Respiratory System |  | 15(2.6) | 6(4.8) | 3(1.3) | 6(2.9) |
| Rheumatology |  | 12(2.1) | 0(0.0) | 1(0.4) | 11(5.2) |
| Sport Sciences |  | 2(0.4) | 0(0.0) | 2(0.8) | 0(0.0) |
| Surgery |  | 26(4.6) | 12(9.6) | 8(3.4) | 6(2.9) |
| Urology & Nephrology |  | 17(3.0) | 6(4.8) | 8(3.4) | 3(1.4) |
| Virology |  | 4(0.7) | 3(2.4) | 0(0.0) | 1(0.5) |
| Values are presented as a number (percentage). | | | | | |
| ^a^ The “Others” category includes clinical trials excluding the phase of drug development. | | | | | |
| ^b^ The “Others” category includes clinical trials herein: trimodality therapy, chemoradiotherapy and surgery, chemoradiation therapy, carbon ion radiation therapy with chemotherapy, radiation therapy, intensified and conventional therapy, brain–machine interface, exercise, psychiatric intervention, and cognitive behavioral therapy. | | | | | |

**
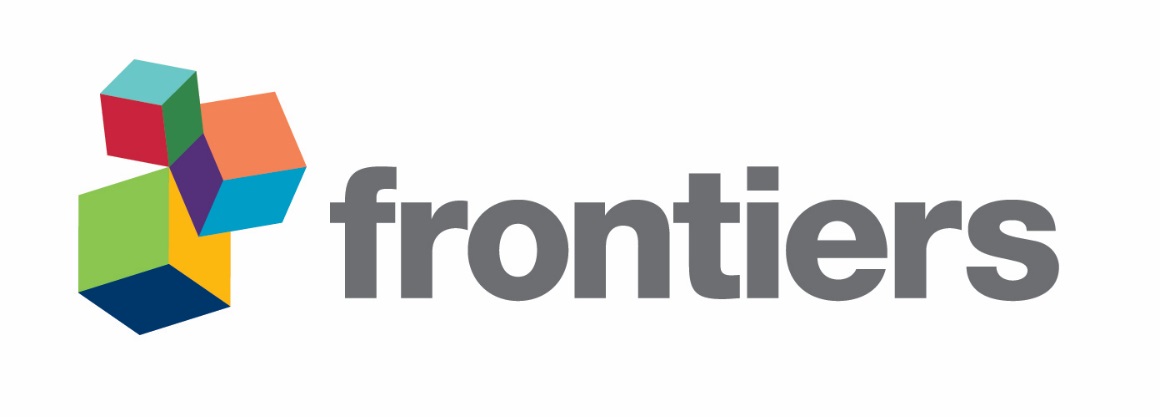
**
